# Supplementary material for: Factors associated with unsuppressed viremia in women living with HIV on lifelong ART in the multi-country US-PEPFAR PROMOTE study: A cross-sectional analysis
Source: PLoS One. 2019 Oct 24;14(10):e0219415. doi: 10.1371/journal.pone.0219415 (PMC6812809; doi:10.1371/journal.pone.0219415)
Supplement: S3 Table — (DOCX) [file pone.0219415.s003.docx]

S3 Table A: Individual and contextual baseline characteristics

| **Variable** | **Viral load**  **≤1000**  **copies/ml (N=1768)** | **Viral load >1000 copies/ml (N=166)** | **Total** | **p-value** |
| --- | --- | --- | --- | --- |
| **Socioeconomic and demographic factors** |  |  |  |  |
| *Country, n (%)* |  |  |  | <0.001 |
| Uganda | 327 (92.9%) | 25 (7.1%) | 352 |  |
| Malawi | 543 (85.8%) | 90 (14.2%) | 633 |  |
| Zimbabwe | 417 (93.1%) | 31 (6.9%) | 448 |  |
| South Africa | 481 (96.0%) | 20 (4.0%) | 501 |  |
| Age (years), median(IQR) | 31 (28 - 35) | 29 (25 - 33) | 31  (27-35) | <0.001 |
| Baseline CD4 cell count (cells/µL), median (IQR) | 848  (672-1063) | 594  (442-768) | 825  (646-1040) | <0.001 |
| *Marital status, n(%)* |  |  |  | 0.002 |
| Other (single, divorced, widowed, separated) | 336 (87.3%) | 49 (12.7%) | 385 |  |
| Married/regular partner | 1432 (92.4%) | 117 (7.6%) | 1549 |  |
| *Employment, n(%)^a^* |  |  |  | 0.056 |
| Formal employment | 397 (93.9%) | 26 (6.1%) | 423 |  |
| Self-employment (small business) | 551 (89.6%) | 64 (10.4%) | 615 |  |
| Not employed/housewife | 818 (91.5%) | 76 (8.5%) | 894 |  |
| *Highest level of education, n(%)* |  |  |  | 0.006 |
| Secondary school completed or tertiary education | 1262 (92.6%) | 101 (7.4%) | 1363 |  |
| Lower level of education | 506 (88.6%) | 65 (11.4%) | 571 |  |
| *Electricity in the premises, n(%)* |  |  |  |  |
| Available | 1244 (94.5%) | 73 (5.5%) | 1317 | <0.001 |
| Not available | 524 (84.9%) | 93 (15.1%) | 617 |  |
| *Tap water in the premises, n(%)* |  |  |  |  |
| Available | 1173 (93.1%) | 87 (6.9%) | 1260 | <0.001 |
| Not available | 595 (88.3%) | 79 (11.7%) | 674 |  |
| *Travel time from home to clinic, n(%)^b^* |  |  |  | 0.113 |
| Less than 30 minutes | 452 (94.0%) | 29 (6.0%) | 481 |  |
| 30-60 minutes | 785 (91.0%) | 78 (9.0%) | 863 |  |
| 1-2 hours | 400 (89.7%) | 46 (10.3%) | 446 |  |
| Greater than 2 hours | 130 (90.9%) | 13 (9.1%) | 143 |  |
| *Disclosed HIV status to partner, n(%)^c^* |  |  |  | 0.677 |
| Disclosed to partner | 1233 (92.6%) | 99 (7.4%) | 1332 |  |
| No disclosure | 199 (91.7%) | 18 (8.3%) | 217 |  |
| *Partner’s HIV status^d^* |  |  |  |  |
| Positive | 746 (92.9%) | 57 (7.1%) | 803 | 0.894 |
| Negative | 265 (92.7%) | 21 (7.3%) | 286 |  |
| *Condom usage during sex in last 3 months, n(%)^e^* |  |  |  | 0.402 |
| Always | 519 (92.8%) | 40 (7.2%) | 559 |  |
| Sometimes | 520 (91.1%) | 51 (8.9%) | 571 |  |
| Never | 270 (90.6%) | 28 (9.4%) | 298 |  |
| *Years on ART, median(IQR)* | 2 (1 - 2) | 1 (1 - 2) | 2 (1-2) | <0.001 |
| **Clinical factors** |  |  |  |  |
| *Admitted to hospital in the past 3 months, n(%)* |  |  |  |  |
| Yes | 23 (79.3%) | 6 (20.7%) | 29 | 0.033 |
| No | 1745 (91.6%) | 160 (8.4%) | 1905 |  |
| *Received TB treatment in the last 3 months, n(%)* |  |  |  |  |
| Yes | 8 (88.9%) | 1 (11.1%) | 9 | 0.555 |
| No | 1760 (91.4%) | 165 (8.6%) | 1925 |  |
| *Presence of abnormal vaginal discharge in the last 3 months, n (%)* |  |  |  |  |
| Yes | 87 (89.7%) | 10 (10.3%) | 97 | 0.575 |
| No | 1681 (91.5%) | 156 (8.5%) | 1837 |  |
| *Currently breastfeeding, n(%)^b^* |  |  |  |  |
| Yes | 169 (88.0%) | 23 (12.0%) | 192 | 0.079 |
| No | 1598 (91.8%) | 143 (8.2%) | 1741 |  |
| ***ART related factors*** |  |  |  |  |
| *ART regimen* |  |  |  |  |
| EFV or NVP based | 1040 (89.3%) | 125 (10.7%) | 1165 | <0.001 |
| LPV/r based | 258 (92.1%) | 22 (7.9%) | 280 |  |
| Fixed dose combination | 470 (96.1%) | 19 (3.9%) | 489 |  |
|  |  |  |  |  |
| *Since last scheduled visit, when was the last time you missed any of your ARV doses? n(%)^f^* |  |  |  |  |
| Never missed any doses | 1283 (93.6%) | 87 (6.4%) | 1370 | <0.001 |
| Within the last week | 74 (81.3%) | 17 (18.7%) | 91 |  |
| 1-2 weeks ago | 42 (89.4%) | 5 (10.6%) | 47 |  |
| 2-4 weeks ago | 66 (85.7%) | 11 (14.3%) | 77 |  |
| 1-3 months ago | 195 (91.5%) | 18 (8.5%) | 213 |  |
| More than 3 months ago | 64 (81.0%) | 15 (19.0%) | 79 |  |
| Don't know or cannot remember | 34 (94.4%) | 2 (5.6%) | 36 |  |
| *Number of days ARV doses missed in last four days, n(%)^f^* |  |  |  |  |
| None | 1682 (93.1%) | 125 (6.9%) | 1807 | <0.001 |
| One day | 53 (88.3%) | 7 (11.7%) | 60 |  |
| Two days | 8 (66.7%) | 4 (33.3%) | 12 |  |
| Three days | 1 (50.0%) | 1 (50.0%) | 2 |  |
| Four days | 12 (46.2%) | 14 (53.8%) | 26 |  |
| Don't know or cannot remember | 2 (33.3%) | 4 (66.7%) | 6 |  |
| *Awareness of dosing instructions, n(%)^f^* |  |  |  |  |
| Yes | 851 (90.5%) | 89 (9.5%) | 940 | 0.029 |
| No | 865 (92.8%) | 67 (7.2%) | 932 |  |
| Don't know or cannot remember | 42 (100.0%) | 0 | 42 |  |

^a^2 participants had missing data, ^b^1 participant had missing data, ^c^amongst 1549 women with partners, ^d^amongst women whom their partner’s got tested and women knew their HIV status,

^e^amongst women who report sexual activities, ^f^amongst those who were on ART at enrollment

Please note that we presented row percentages to display the proportion of patients with detectable viremia for each level of the variable.

S3 Table B: Factors associated with detectable viral load >1000 copies/ml

|  | **Multivariable^1^** | | **Multivariable^2^** | |
| --- | --- | --- | --- | --- |
| **Variable** | **RR (95% CI)** | **p-value** | **aRR (95% CI)** | **p-value** |
| Age (5-year increase) | 0.76 (0.65-0.89) | <0.001 | 0.76 (0.64-0.89) | <0.001 |
| Marital status (ref: married/regular partner) | | | | |
| Other (single, divorced, widowed, separated) | 1.69 (1.24-2.30) | <0.001 | 1.35 (0.96-1.89) | 0.086 |
| Employment (ref: formal employment) | | | | |
| Not employed | 1.16 (0.75-1.78) | 0.510 | 1.01 (0.65-1.57) | 0.976 |
| Self employed | 1.26 (0.80-2.00) | 0.319 | 1.27 (0.79-2.03) | 0.321 |
| Education (ref: secondary school not complete) | | | | |
| Secondary school complete | 0.99 (0.72-1.38) | 0.968 | 1.22 (0.85-1.75) | 0.279 |
| Electricity in the premises (ref: Yes) | | | | |
| No | 2.04 (1.47-2.84) | <0.001 | 2.19 (1.52-3.17) | <0.001 |
| Tap water in the premises (ref: Yes) | | | | |
| No | 1.41 (1.04-1.91) | 0.026 | - | - |
| Travel time to clinic from home (ref: less than 1 hour) | | | | |
| 1 hour or more | 1.07 (0.78-1.47) | 0.672 | 0.93 (0.66-1.31) | 0.685 |
| Condom use during sex in last three months (ref: Never) | | | | |
| Always | 0.93 (0.57-1.51) | 0.764 | - | - |
| Sometimes | 0.95 (0.62-1.47) | 0.830 | - | - |
| HIV status disclosure to partner (ref=Yes) | | | | |
| No | 1.42 (0.85-2.38) | 0.178 | - | - |
| Abnormal vaginal discharge in last three months (ref: No) | | | | |
| Yes | 1.62 (0.87-3.04) | 0.132 | 1.42 (0.76-2.65) | 0.266 |
| Hospital admission in last three months (ref: No) | | | | |
| Yes | 2.58 (1.24-5.37) | 0.011 | 2.85 (1.32-6.14) | 0.008 |
| Aware of ARV medication dosing instructions (ref: No) | | | | |
| Yes | 1.07 (0.77-1.49) | 0.685 | 1.09 (0.78-1.52) | 0.612 |
| Missed ART doses since last visit (ref: None) | | | | |
| Missed some doses | 1.97 (1.46-2.66) | <0.001 | 1.59 (1.16-2.18) | 0.004 |
| Time since ART initiation  (per 1-year increase) | 0.61 (0.49-0.75) | <0.001 | 0.67 (0.56-0.81) | <0.001 |

^1^Each predictor fitted separately while adjusted for the country ^2^Multivariable model with multiple predictors
