# Supplementary material for: Factors associated with unsuppressed viremia in women living with HIV on lifelong ART in the multi-country US-PEPFAR PROMOTE study: A cross-sectional analysis
Source: PLoS One. 2019 Oct 24;14(10):e0219415. doi: 10.1371/journal.pone.0219415 (PMC6812809; doi:10.1371/journal.pone.0219415)
Supplement: Attachment — Submitted filename: Comments.docx [file pone.0219415.s004.docx]

The authors conducted an analysis to explore factors associated with viremia above 200 among women from multiple sites in Africa. I found the paper to be written well with a clear logical flow and tight focus. However, I have identified minor concerns that the authors should work on.

- Page 11, line 87 …are most strongly assoicated with 87 unsuppressed…..

Correct the typo

- Font size is not consistent, see beginning of results section, and probably other places
- Footnote in table2

a2 missing data, b1 missing data, camongst 1549

What do these numbers 2 and 1 supposed to represent?

- Reference 2, 2018 [ Is there something missing, it appears as if you started unclosed bracket?
