# Supplementary material for: Factors associated with unsuppressed viremia in women living with HIV on lifelong ART in the multi-country US-PEPFAR PROMOTE study: A cross-sectional analysis
Source: PLoS One. 2019 Oct 24;14(10):e0219415. doi: 10.1371/journal.pone.0219415 (PMC6812809; doi:10.1371/journal.pone.0219415)
Supplement: Attachment — Submitted filename: revision plos one paper.docx [file pone.0219415.s005.docx]

Please address the following major issues that need addressed before the paper could be published.

**Title**

1) Reading the title can be misleading in that it would make a reader think that you are performing a cohort study. Please reformulate to make the study design of the present analysis clear (which I think is cross-sectional), rather than focusing on the parent study.

**Abstract**

2) The designation “Africa women” may be misleading. It may be too general given that your sample is comprised of women from Eastern and Southern Africa. Countries such as Egypt and Libya are parts of Africa and if you believe that your findings apply to these countries, please elaborate so. However, please reformulate all instances of the word “Africa” to be more specific to your population of interest.
3) Please include the design of your analysis in the abstract.
4) In line 42, did you mean “to assess”
5) Please include the measure of association that you aimed to estimate in the method section.
6) The conclusion reads “Socioeconomic barriers such as poverty, not being married, young age, and self-reported missed doses remain key predictors of unsuppressed viremia.” I’d suggest restructuring the sentence because it seems that “not being married, young age…” are part of the socioeconomic barriers.
7) The word “remain” in the conclusion can be misleading because the present analysis is not longitudinal in nature. If you want to refer to a previous publication of the same group, please elaborate so.

**Introduction**

8) Line 66, please update the statistics about the number of people receiving cART, to a more recent date. Your statistics are from 2017.
9) Line 70, “ … 90-90-90 2020 Strategy in ending the epidemic by 2030 (2).” Does not read well. There is a capital letter in the middle of sentence.

10) Line 87. Please correct the word “assoicated”
11) Line 90. Suggest not starting “Tuberculosis” with capital letter.
12) Please provide a relevant reference to the following sentence “Emerging antriretroviral drug resistance has been known to occur from levels of 200 copies/ml or above, and use of this threshold eliminates most cases of apparent viremia caused by viral load blips or assay variability”

The WHO considered VL>1000 as threshold to define viral failure, to reduce pitfalls from viral blips. If you believe that VL<200 eliminates most viral blips, please provide the reference supporting this.

**Method**

13) Please focus on the description of the design of this present study. You were focusing on describing the parent study PROMOTE.
14) Please indicate what was defined as “high enrolling PROMISE sites”
15) Please briefly describe the intervention in PROMISE study, to relieve any concern that the PROMISE intervention could have an impact on viremia among women in your study sample.
16) By definition, exclusion criteria are characteristics of subjects who have met the inclusion criteria. Therefore, if “willing to provide informed consent to enroll and continue follow-up” is an inclusion criterion, it is would be redundant to write “unwilling to provide informed consent to continue follow-up” as exclusion criterion, because both represent the same thing. If you meant to say that participants who refused to provide consent after being enrolled are excluded, I would suggest making it clearer.
17) Please be specific about criteria used to make the judgment in line 132
18) Please add in the method section a description of covariates: how they were defined and coded. In addition, please indicate the justification for each coding scheme. If your group has used the coding in a previous manuscript, please provide relevant citation.
19) Could you add information about the testing procedure: blood specimen manipulation, storage, type of laboratory, existing quality control.
20) I do not think that the following sentence is relevant to your present objectives “Samples were stored for future HIV drug resistance testing (blood) and cumulative drug levels testing (hair).”
21) Please clearly indicate why did you use the Poisson regression and why you did not use log binomial regression.
22) You mentioned that you used prevalence risk ratio as measure of association. However, in the abstract you mentioned that you used prevalence rate ratio. I would suggest being consistent.
23) You wrote that each predictor was fitted in the model as first step of your modeling(line 159). However, in line 155 it reads “Fisher’s exact, Chi-square test of independence or Wilcoxon rank sum tests were used to test for an association between baseline characteristics and unsuppressed viremia”

Which one was used in the univariable analyses ?

24) “and (ii) multivariable model with all the predictors included in the model” This sentence has no verb
25) It seems as if you are contradicting yourselves by including the following phrases in the method section “…. multivariable model with all the predictors included in the model” AND “Variables included in the multivariable analyses were chosen based on prior research on risk factors, biological plausibility, and previously identified clinical associations”. The ALL in the first sentence may be understood as you included all covariates from the univariable analyses in the multivariable model.
26) The following sentence “… prior research on risk factors, biological plausibility, and previously identified clinical associations”

Using “Prior research” and “previously identified clinical associations” in the same sentence is redundant. In addition, please provide citation for the previously identified clinical associations.

**Results**

27) Please provide in the supplemental materials the results using VL>1000 as secondary outcome.
28) Please include a flowchart or a relevant table that summarizes the following information “Overall, 1987 mothers were enrolled into the PROMOTE study, of whom 1947 (98%) women reported taking ART at the enrolment visit and HIV-1 viral load results were available for 1934. HIV-1 VL was above the limit of quantification in 293/1934 (15 %). ”
29) Table 1 does not provide with an overall description of the sample, because it is stratified by viral suppression status. Please add a third column containing an overall frequency.
30) The notations p-values are not consistent, sometimes they are written as “<.001” . Sometimes, they are written as “<0.001”
31) For each association you describe in the result, please include the comparison group.
32) Please be consistent in the notation of copies/ml. Sometimes you use cp/ml, sometimes you use copies/ml
33) “In addition, the absence of socioeconomic factors such as electricity in the premises” The sentence does not read well. Which premises ?
34) Did you mean “travel time from the cART clinic “ or “travel time to the cART clinic “
35) I do not think that the following sentence is relevant with regards to your study objective. “Additionally, about 10% (n=29) of women with unsuppressed viremia had an HIV-uninfected partner.”

**Discussion**

36) The following sentence does not read well. “We identify sociodemographic, self-reported non-adherence, and clinical factors that were associated with detectable viremia > 200copies/ml.”

37) LINE 230, you used “correlation” …. Correlation analyses and regression analyses are very different.

38) Line 292: The longitudinal design of the PROMOTE does not confer any strength to this present analysis, which is a cross-sectional analysis of the baseline data of a longitudinal analysis. However, the sample size is a strength. Please reformulate.

39) Please elaborate more on the ongoing quality assurance that would strengthen this present cross-sectional analysis.

40) There are additional limitations that ought to be discussed: potential recall bias due to self-report, the inability to confirm viral failure.
